# Value of high frame rate contrast-enhanced ultrasound in predicting microvascular invasion of hepatocellular carcinoma

**DOI:** 10.1186/s13244-024-01821-6

**Published:** 2024-11-15

**Authors:** Xiang Fei, Lianhua Zhu, Peng Han, Bo Jiang, Miao Li, Nan Li, Ziyu Jiao, Dirk-André Clevert, Yukun Luo

**Affiliations:** 1 Department of Ultrasound, The First Medical Centre, Chinese PLA General Hospital, Beijing, China; 2 Interdisciplinary Ultrasound-Center University of Munich-Grosshadern Campus, Munich, Germany

**Keywords:** Hepatocellular carcinoma, Vascular morphology, High frame rate contrast-enhanced ultrasound, Recurrence, CEUS Li-RADS

## Abstract

**Objectives:**

To investigate the value of vascular morphology on high frame rate contrast-enhanced ultrasound (H-CEUS) and CEUS Li-RADS in predicting microvascular invasion (MVI), Ki-67 expression and recurrence of hepatocellular carcinoma (HCC).

**Methods:**

This retrospective study enrolled 78 patients with single HCC diagnosed by postoperative pathology between January 1, 2021, and June 30, 2022. All patients underwent ultrasound and H-CEUS examination before operation. H-CEUS image features and CEUS Li-RADS were compared in different MVI status and Ki-67 level. Multiple logistic regression analysis was performed to select independent variables for MVI. Differences in recurrence among different H-CEUS image features, MVI status and Ki-67 level were further analyzed.

**Results:**

Tumor shape, vascular morphology, LR-M category, necrosis and AFP level were different between the MVI-positive group and MVI-negative group (*p* < 0.05). Vascular morphology and LR-M category were independent risk factors related to MVI (*p* < 0.05). Vascular morphology was also different between the high Ki-67 expression group and low Ki-67 expression group (*p* < 0.05). Vascular morphology, MVI status and Ki-67 expression were different between the recurrence group and no recurrence group (*p* < 0.05).

**Conclusion:**

The vascular morphology of HCC on H-CEUS can indicate the risk of MVI status, Ki-67 expression and recurrence, which provides a feasible imaging technique for predicting the prognosis before operation.

**Critical relevance statement:**

H-CEUS shows the different vascular morphology of HCC in arterial phase and indicates the risk of MVI, Ki-67 expression and recurrence, which provides a feasible imaging technique for clinician to judge the risk of MVI pre-operation and adopt appropriate treatment.

**Key Points:**

- H-CEUS can clearly show different vascular morphology of HCC in arterial phase.
- Vascular morphology on H-CEUS is associated with MVI status, Ki-67 expression and HCC recurrence.
- Preoperative MVI and Ki-67 expression prediction could help surgeons choose a more appropriate treatment plan.

**Graphical Abstract:**

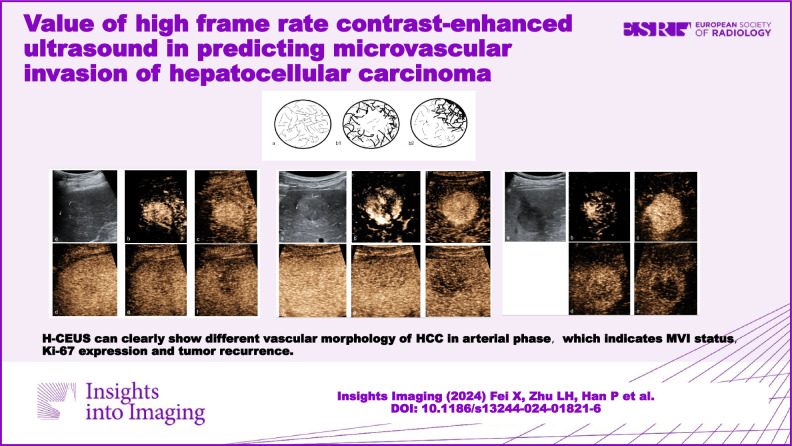

## Introduction

Hepatocellular carcinoma (HCC) is the most common primary liver malignant epithelial tumor derived from hepatocytes with a high morbidity, mortality rate and poor prognosis around the world [1–5]. Currently, liver resection and transplantation are the possible curative treatments for HCC [6, 7]. However, tumor recurrence rate after surgery is up to 70%, and 5-year recurrence-free survival is as low as 26–40% [8–10]. Although the poor prognosis of HCC is multifactorial, microvascular invasion (MVI) has been reported to serve as the independent risk factors related to tumor recurrence [11, 12], because MVI is an indicator of more aggressive tumor biology. Therefore, accurate prediction and evaluation of MVI are critical for making appropriate treatment strategies before surgery.

MVI exists mainly in small blood vessels such as portal vein branches, and it can be accurately detected only by postoperative histopathological examination [13, 14]. Currently, there is no preoperative imaging technique that can directly diagnose MVI. Contrast-enhanced ultrasound (CEUS) has shown a promising clinical application in the prediction of MVI. Some CEUS features were independent risk factors related to MVI, such as rapid wash-out, enhancement pattern in portal venous and delayed phase, and tumor necrosis [15, 16]. Another study reported that none of the qualitative CEUS features were independently associated with MVI [17]. Therefore, it is necessary to further explore the correlation between CEUS features and MVI.

Development of its arterial blood supply and sinusoidal capillarization are the two most important features of HCC [18]. The arterial perfusion of HCC could be displayed by CEUS. Therefore, the relationship between neoplastic vessels changes and different enhancement pattern of various differentiation HCC could be detected by CEUS [19]. CEUS features in arterial phase (AP) might be associated with MVI. However, no studies have reported that CEUS features in AP are independent risk factors related to MVI. We thought that CEUS technique used in the previous research has some limitations in the temporal resolution of the images, which made it difficult to accurately reflect the perfusion characteristics in AP, as a probable result, no correlation was found between CEUS features in AP and MVI. H-CEUS improves the temporal resolution by increasing the frame rate, which is conducive to the observation of the details of blood perfusion of small lesions rich in blood supply. In our previous study, we found that H-CEUS could accurately reflect the AP perfusion features of focal hepatic lesions (FLL), especially to reflect vascular morphology of FLL compared to conventional CEUS [20]. Therefore, we speculated that H-CEUS may be able to overcome the limitation of CEUS and reflect the correlation between vascular morphology in AP and MVI.

Besides, Ki-67 protein is associated with cell proliferation, and higher Ki-67 expression confers a fast progression and poor prognosis in HCC [21–23]. However, it is difficult to directly evaluate Ki-67 levels by current preoperative imaging techniques. H-CEUS imaging features may be associated with the expression of Ki-67 in HCC. To the best of our knowledge, no studies have yet used H-CEUS to explore the relation between vascular morphology in AP and MVI status and Ki-67 expression. In addition, CEUS Li-RADS has been widely applied in distinguishing FLL, while few studies have explored the value of CEUS Li-RADS in predicting MVI status and Ki-67 expression.

In this study, we aimed to explore vascular morphology features of HCC in AP by H-CEUS, investigate the correlation between vascular morphology combined with CEUS Li-RADS and MVI status and Ki-67 expression, and evaluate the risk factors related to MVI, which could provide more diagnostic information for the physician to select an appropriate method.

## Materials and methods

### Patients

**Fig. 1:**
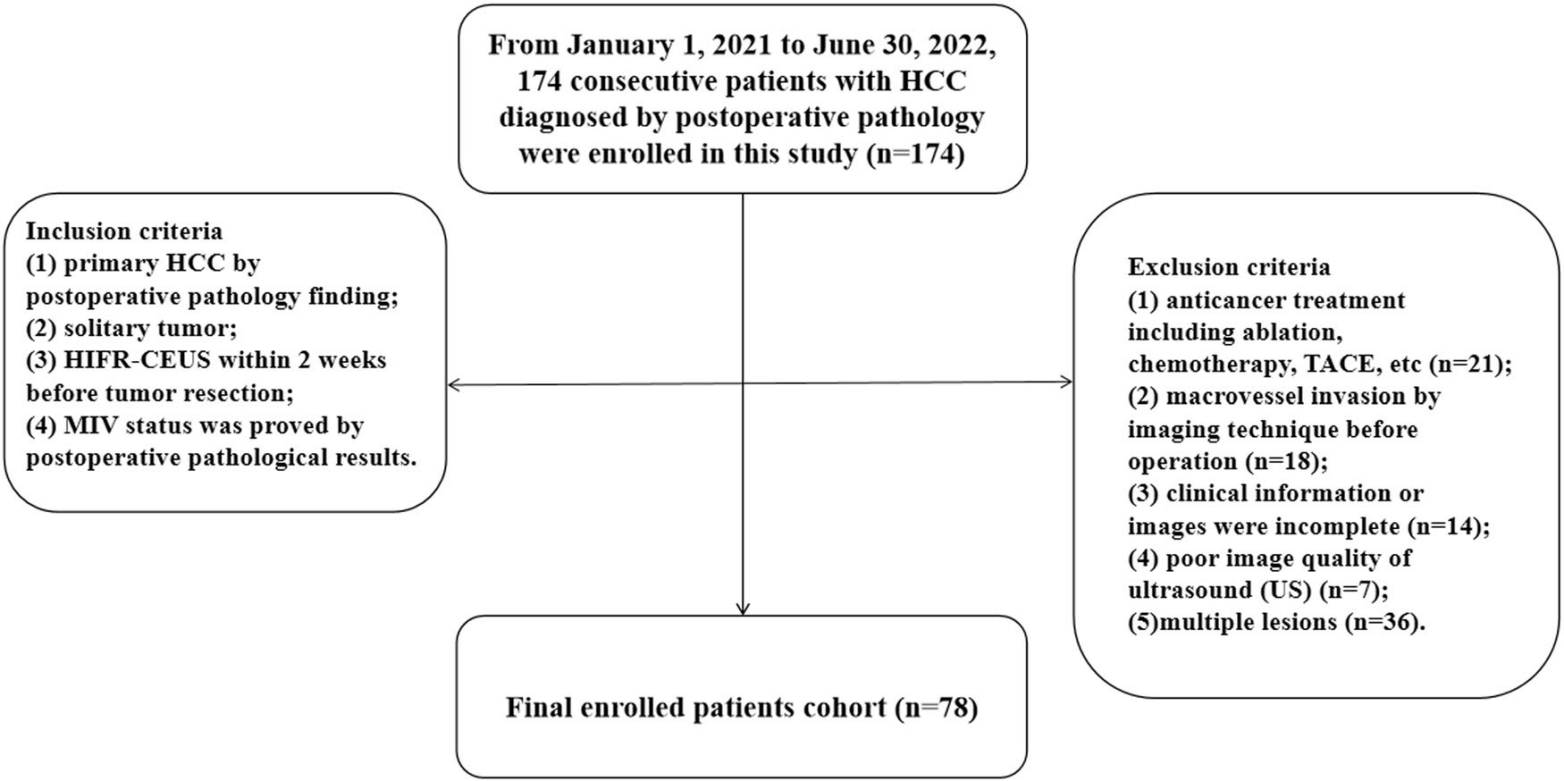
Flowchart of patient enrolment. HCC, hepatocellular carcinoma; H-CEUS, high frame rate contrast-enhanced ultrasound

This study was approved by the Ethics Committees of the Chinese PLA General Hospital. Between January 1, 2021, and June 30, 2022, 174 consecutive patients with HCC diagnosed by postoperative pathology were enrolled in this study. All enrolled patients were followed up, and the median follow-up duration was 13 months after operation. Follow-up protocol was as follows: after curative resection, the patients were followed up with dynamic enhanced computed tomography, magnetic resonance imaging, ultrasonography, serum Alpha-fetoprotein (AFP) levels, blood routine examination, liver function and kidney function. The postoperative follow-up evaluation was performed 1 month after resection, every 3 months in the first 2 years. The inclusion criteria were as follows: (1) primary HCC by postoperative pathology finding; (2) solitary tumor; (3) H-CEUS within 2 weeks before tumor resection; (4) MVI status was proved by postoperative pathological results. The exclusion criteria were as follows: (1) anticancer treatment including ablation, chemotherapy, transcatheter arterial chemoembolization (*n* = 21); (2) macrovessel invasion by imaging technique before operation (*n* = 18); (3) clinical information or images were incomplete (*n* = 14); (4) poor image quality of ultrasound (US) (*n* = 7); (5) multiple lesions (*n* = 36). According to the inclusion and exclusion criteria, in total, 78 cases of HCC were finally enrolled in this study, and Fig. 1 displays the flowchart of study patients.

### Clinical information and pathology findings

Clinical information, including age, gender, family history of HCC and hepatitis serology, was obtained from medical records. Preoperative serum examination, including platelets (ALT), albumin (ALB), AFP, were measured before surgery within 2 weeks. Pathological findings included MVI status, Ki-67 expression and differentiation of HCC (well, moderate, or poor according to the WHO histologic grade system). Specimens were evaluated by a pathologist blinded to clinical features and H-CEUS data. MVI was defined as the presence of tumor emboli in a vascular space lined by endothelium that can only be observed under microscopy. Ki-67 expression was assessed by counting the frequency of Ki-67 positive cell. According to previous studies, immune-reactive cells were classified as either low Ki-67 expression (≤ 10% immunoreactivity) or high Ki-67 expression (> 10% immunoreactivity) [24, 25]. The recurrence criteria were as follows: The diagnosis of intrahepatic recurrence based on imaging alone (at least 2 radiological imaging modalities), if the tumor displayed the typical enhancement characteristics. Those with atypical imaging characteristics were biopsied to confirm HCC. Extrahepatic tumors were biopsied to confirm metastasis.

### H-CEUS and US examination

US equipment used to perform US and H-CEUS scanning in this study was Mindray Resona 9 (Mindray, Shenzhen, China) The transducer used was C5-1 (frequency 2–5 MHz). The H-CEUS software was ultra-wide nonlinear, and the frame rate could be from 36 to 60 Hz. A low mechanical index ranging from 0.06 to 0.09 was used for real-time H-CEUS. The contrast agent used was SonoVue (Bracco, Milan, Italy). US and H-CEUS were performed by a single ultrasound doctor who had more than 10 years of experience in abdominal CEUS.

### Features of US and H-CEUS images

**Fig. 2:**
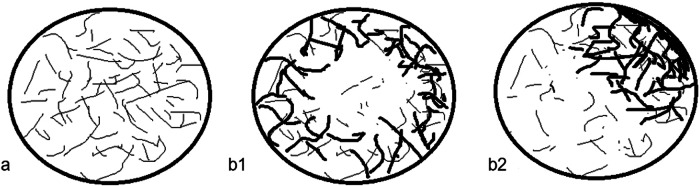
Diagram of vascular morphology type of HCC. **a** Type 1: irregular vessels with dense and homogeneously distributed within the lesion. **b** Type 2: large vessels in the periphery of the tumor that are sparsely distributed within the tumor

First, US was performed to evaluate tumor size, echoic level (hyper-echoic, iso-, and hypo, compared to adjacent liver parenchyma), boundary (clear, unclear), shape (regular, irregular), and vascularity by color Doppler flow imaging (CDFI) (present, absent). H-CEUS features were as follows: (1) enhancement intensity in AP (hyper-enhancement, non-hyper, compared to adjacent liver parenchyma); (2) vascular morphology of HCC in AP: type 1 defined as fine irregular vessels with dense and homogeneously distributed within the lesion, type 2 defined as relatively large vessels in the periphery of the tumor that are either absent or sparsely distributed within the tumor (Fig. 2); type 3: unidentified vascular morphology, which was different from type 1 and type 2; (3) necrosis within HCC in AP (absent or present); and (4) CEUS Li-RADS (LR) M (Yes or No, LR M means a tumor shows rim enhancement in AP, or early wash-out or marked wash-out) [26].

Two ultrasound doctors who had more than 15 years of experience in abdominal CEUS independently interpreted the US and H-CEUS features [27–29]. Each doctor was blinded to patient clinical information and pathology findings. Consistency between the two doctors was assessed with analysis of inter-observer agreement test.

### Statistics

Statistical analysis was performed with SPSS 21.0 (IBM Corporation, Armonk, NY, USA). All quantitative parameters are expressed as mean ± standard deviation. The Student’s t-test was used to compare the differences in continuous variables between the two groups. Comparisons of categorical data were performed using the χ^2^ test or Fisher’s exact test. Univariate analysis was used to identify risk factors. Variables with *p* < 0.05 in univariate analysis were included in multivariate logistic regression analysis. Multivariate logistic regression analysis was used to assess independent risk factors with MVI. The concordance of agreement between observers was tested with Cohen’s Kappa test. A *p*-value less than 0.05 was considered statistically significant.

## Results

### General information of patients

The total of 78 patients’ clinical information and pathology findings are in Table 1. The HCC patients enrolled are all single lesions, including 21 solitary lesions ≤ 2 cm in diameter (very early stage, BCLC-0) and 57 solitary lesions > 2 cm in diameter (early stage, BCLC-A). Most patients had Hepatitis B or C. Of 78 patients, 62 had liver cirrhosis proved by pathology. The number of well, moderate and poor differentiation HCC were 19 (24.3%), 46 (59.0%), and 13 (16.7%), respectively. MVI was proved by postoperative pathology findings in 29.5% cases of HCC. Forty cases of HCC were small lesion which maximum size ≤ 3.0 cm. Sixteen cases were recurrence and 62 cases were no recurrence during follow-up. The coefficients of H-CEUS and US interpretations ranged from 0.77 to 0.86, which meant very good agreement.

**Table 1:** Patients’ general characteristics

Variables	
Age (years)	
Median (range)	59 (32–79)
Sex	
Male	69 (88.5%)
Female	9 (11.5%)
Hepatitis B surface antigen	
Negative	11 (14.1%)
Positive	67 (85.9%)
Hepatitis C	
Negative	72 (92.3%)
Positive	6 (7.7%)
Liver cirrhosis	
Absent	16 (20.5%)
Present	62 (79.5%)
ALT (U/L), median (range)	24.3 (7.3–218.1)
PLT (× 10^9^), median (range)	161.5 (54–492)
AFP (µg/L), median (range)	6.0 (0.6–8139.0)
ALB (g/L), median (range)	41.6 (19.2–50.4)
Tumor size (cm), median (range)	3.2 (1.0–7.0)
Histological differentiation	
Well	19 (24.3%)
Moderate	46 (59.0%)
Poor	13 (16.7%)
Microvascular invasion	
Absent	55 (70.5%)
Present	23 (29.5%)
Ki-67 expression	
Low level (≤ 10%)	32 (41.0%)
High level (> 10%)	46 (59.0%)
Follow-up	
Recurrence	16 (20.5%)
No recurrence	62 (79.5%)

*PLT* platelets, *ALT* alanine aminotransferase, *ALB* albumin, *AFP* alpha-fetoprotein

### H-CEUS features related to MVI

**Fig. 3:**
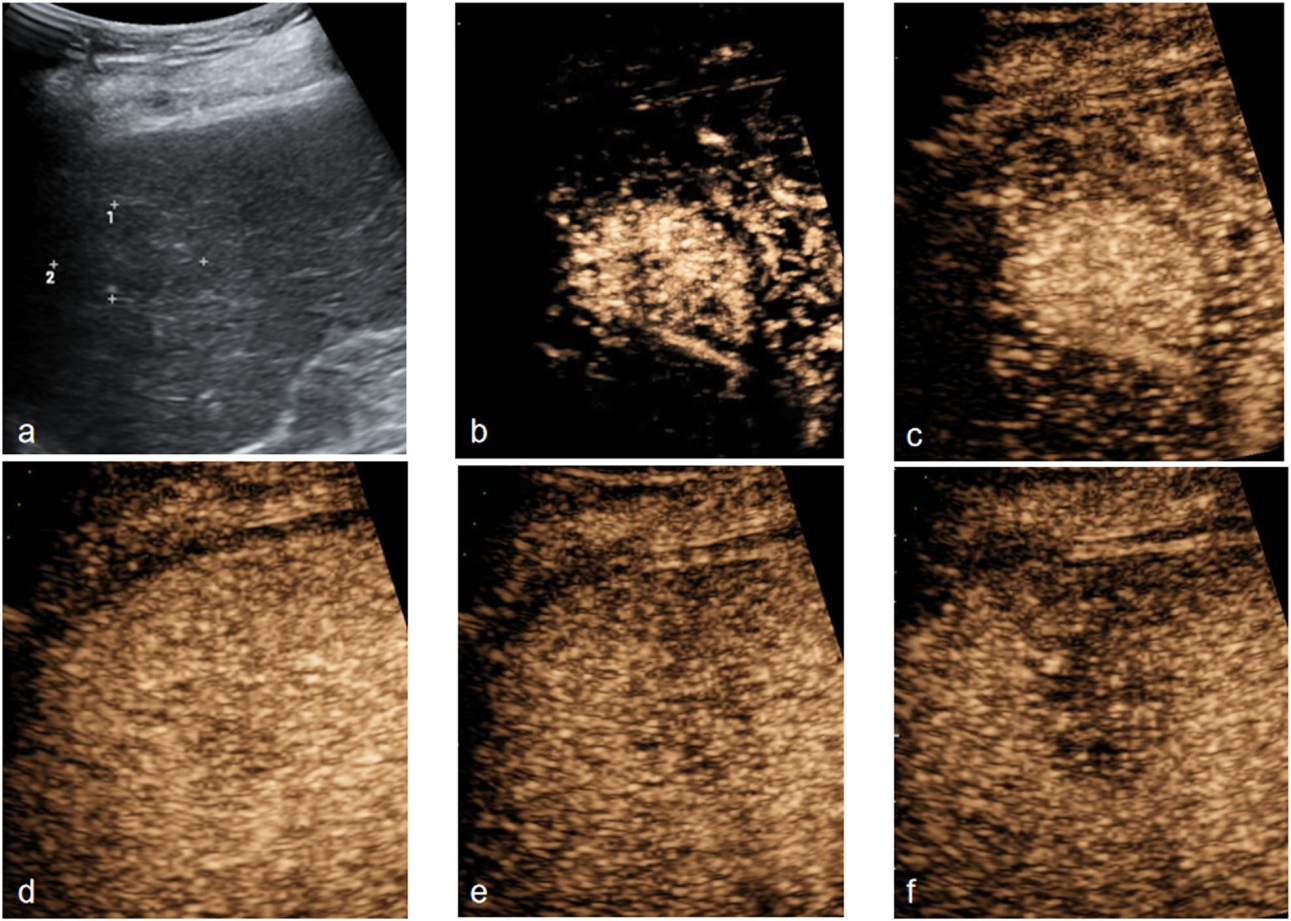
US and H-CEUS images in a 65-year-old female with HCC. **a** Iso-echoic lesion was detected in liver by US. **b** Vascular morphology type 1 in AP by H-CEUS. **c** Homogeneous hyper-enhancement in AP. **d** Iso-enhancement in portal venous phase (60 s). **e** Hypo-enhancement in portal venous phase (90 s). **f** Hypo-enhancement in delayed phase (125 s). Pathology finding proved moderate differentiation HCC without MVI

**Fig. 4:**
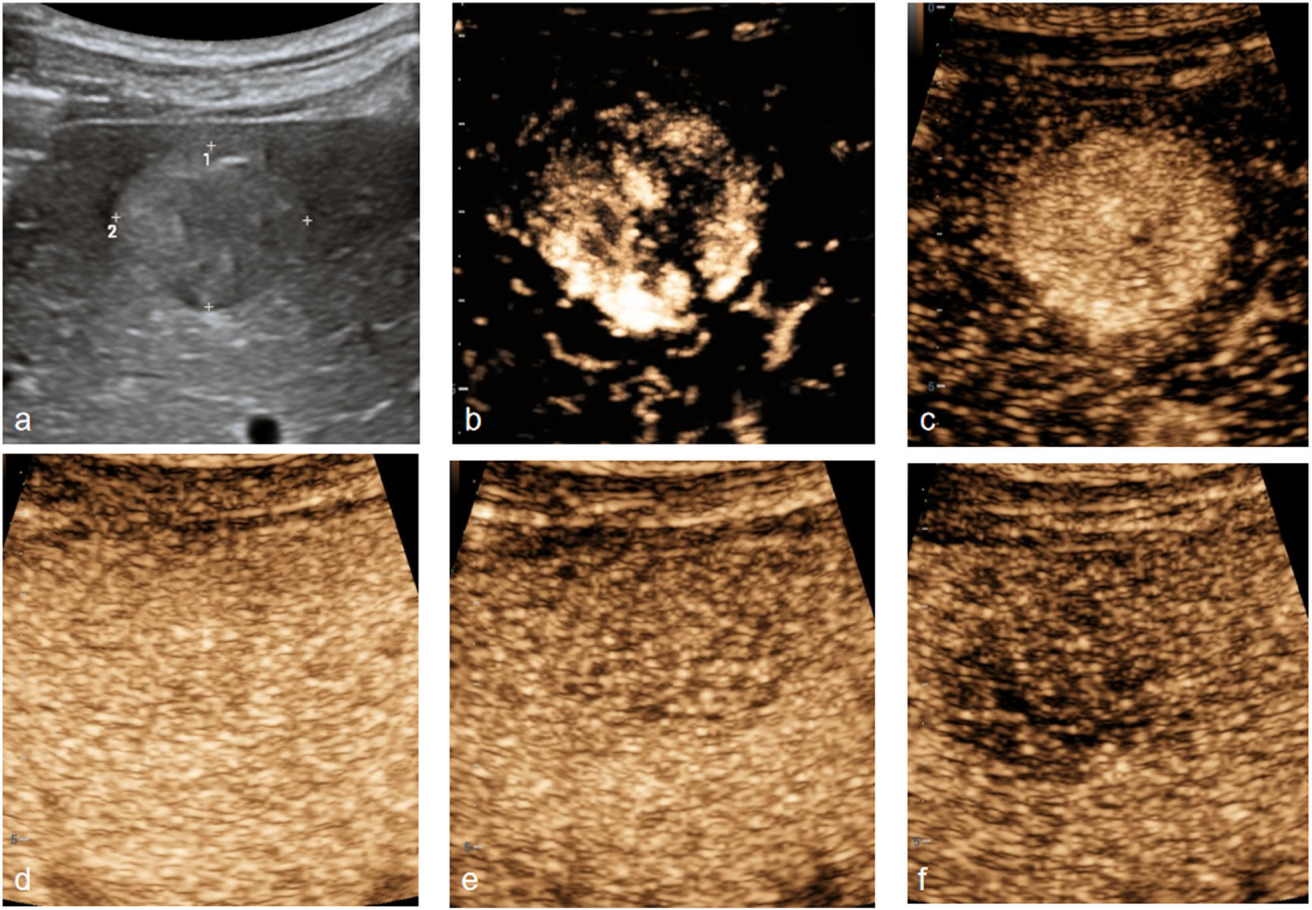
US and H-CEUS images in a 51-year-old male with HCC. **a** Hyper-echoic lesion was detected in liver by US. **b** Vascular morphology type 2 in AP by H-CEUS. **c** Homogeneous hyper-enhancement in AP. **d** Iso-enhancement in portal venous phase (60 s). **e** Mild hypo-enhancement in delayed phase (125 s). **f** Hypo- and no-enhancement in delayed phase (180 s). Pathology finding proved moderate differentiation HCC with MVI

**Fig. 5:**
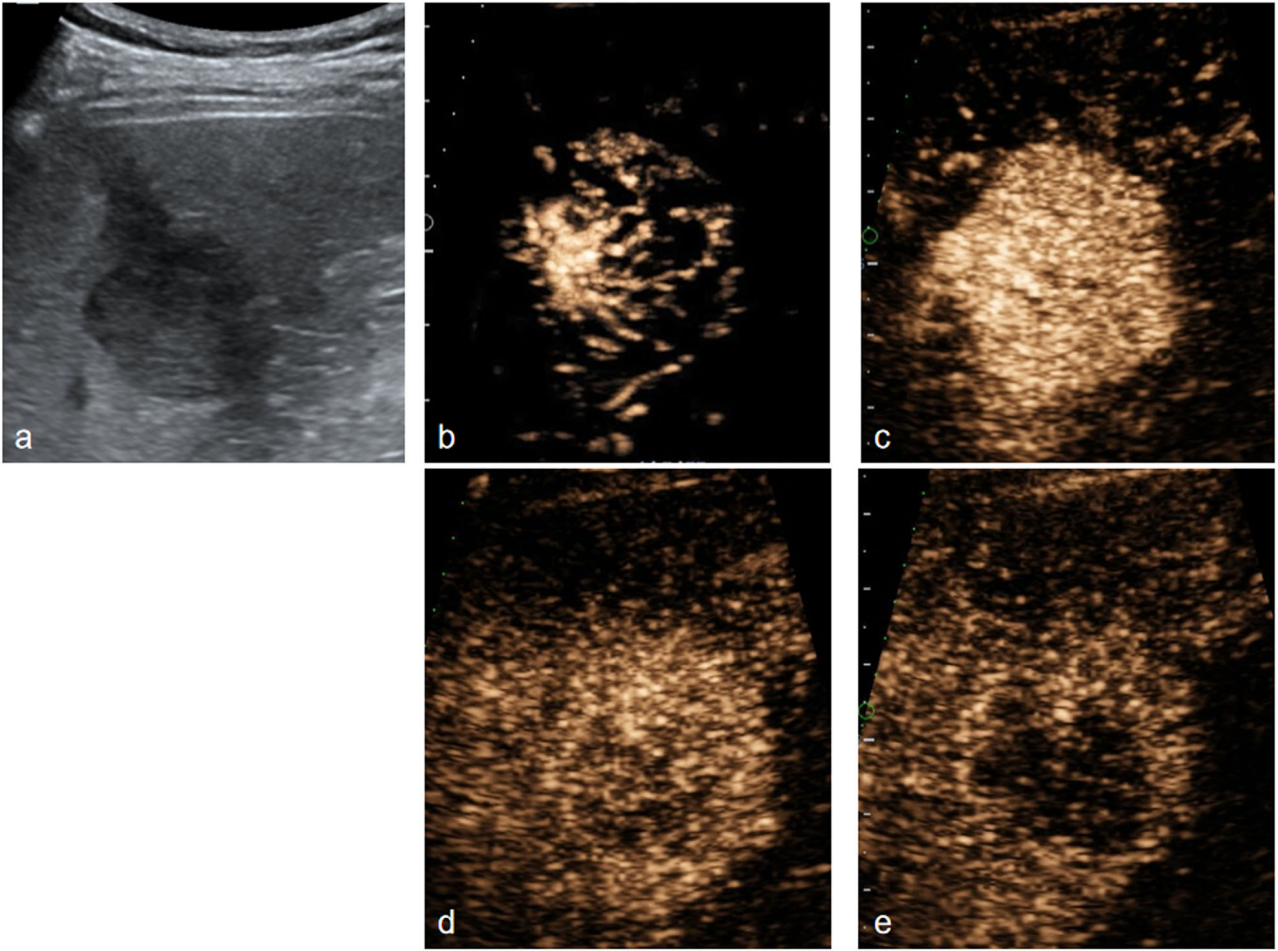
US and H-CEUS images in a 49-year-old male with HCC. **a** Hypo-echoic lesion was detected in liver by US. **b** Vascular morphology type 2 in AP by H-CEUS. **c** Homogeneous hyper-enhancement in AP. **d** Hypo-enhancement in the early portal venous phase (35 s). **e** No enhancement in the portal venous phase (90 s). The lesion enhancement features were coherent with LR-M. Pathology finding proved moderate differentiation HCC with MVI

Univariate analysis found that tumor shape, vascular type, necrosis, AFP and LR-M were statistically different between the MVI-positive group and MVI-negative group (Table 2). In the MVI-positive group, 43.48% cases of HCC were irregular shape. Vascular morphology type 2 was detected in 82.61% cases of HCC with MVI (Fig. 3), while 70.91% cases of HCC without MVI were vascular morphology type 1 (Fig. 4). Necrosis was detected in 60.87% of HCC with MVI, while only 25.45% cases of HCC without MVI had necrosis. The ratio of cases that were in conformity with LR-M in the MVI-positive group was higher than that of in MVI-negative group. In addition, AFP level in the MVI-positive group was higher than that in the MVI-negative group (Table 2). Enhancement intensity in AP, tumor boundary, echoic level of HCC and CDFI characteristics were not statistically different in the MVI-positive group and MVI-negative group (Table 2). For the total of 78 cases of HCC, multiple logistic regression analysis proved that only two variables, including vascular morphology and LR-M (Fig. 5), were independent risk factors related to MVI (Table 3).

**Table 2:** Univariate analysis of risk factors for MVI

Variables	MVI (+) (*n* = 23)	MVI (−) (*n* = 55)	χ^2^/t	*p*-value
Age	59.17 ± 11.84	59.49 ± 10.14	−0.102	0.905
Gender			1.652	0.199
Male	22	47		
Female	1	8		
Tumor size	3.92 ± 2.0	3.76 ± 2.78	0.252	0.802
Boundary			2.518	0.113
Clear	15	45		
Unclear	8	10		
Tumor shape			4.495	0.034
Regular	10	38		
Irregular	13	17		
Echoic level			0.838	0.658
Hyper-	13	25		
Iso	2	7		
Hypo-	8	23		
CDFI			3.216	0.073
Absent	0	7		
Present	23	48		
Enhancement intensity in AP			0.022	0.882
Hyper	22	53		
Non-hyper	1	2		
Vascular morphology in AP			23.354	0.000
Type 1	3	40		
Type 2	20	15		
Type 3	0	0		
Necrosis			8.840	0.003
Absent	9	41		
Present	14	14		
LR-M			14.129	0.000
Yes	9	3		
No	14	52		
AFP (µg/L)	1300.03 ± 2438.55	108.60 ± 317.96	2.341	0.029
ALT (U/L)	50.64 ± 56.12	34.04 ± 34.56	1.592	0.115
ALB (g/L)	41.33 ± 3.04	40.76 ± 4.79	0.507	0.613
PLT (× 10^9^)	162.35 ± 57.81	165.23 ± 67.33	−0.061	0.951

*MVI* microvascular invasion, *AP* arterial phase, *PLT* platelets, *ALT* alanine aminotransferase, *ALB* albumin, *AFP* alpha-fetoprotein, *LR-M* Liver Imaging Reporting and Data System M

**Table 3:** Multivariate Logistic regression analysis of independent risk factors related to MVI

Variables	B	S.E.	Wald	OR	95% CI	*p*-value
Vascular morphology in AP	2.545	0.766	11.027	12.744	2.837–57.240	0.001
LR-M	2.211	0.875	6.393	9.127	1.644–50.668	0.011
Constant	−3.112	0.698	19.880	0.045		0.000

*MVI* microvascular invasion, *CI* confidence interval, *OR* odds ratio, *AP* arterial phase, *LR-M* Liver Imaging Reporting and Data System M

### H-CEUS features related to Ki-67 expression

Patients were divided into two groups according to the expression level of Ki-67. There were differences in vascular morphology type between the high Ki-67 expression group and low Ki-67 expression group (*p* < 0.05) (Table 4). In the high expression group, 52.17% cases were vascular morphology type 2, while in the low expression group, only 25.0% of cases were vascular morphology type 2. LR-M category was not statistically different in the MVI-positive group and MVI-negative group (Table 4).

**Table 4:** Different H-CEUS features and clinical data between Ki-67 high-level group and Ki-67 low-level group

Variables	Ki-67 level (> 10%) (*n* = 46)	Ki-67 level (≤ 10%) (*n* = 32)	χ^2^/t	*p*-value
Age (years)	60.08 ± 11.81	59.13 ± 8.25	0.398	0.692
Gender			0.049	0.825
Male	41	28		
Female	5	4		
Tumor size (cm)	3.76 ± 2.47	3.86 ± 2.72	−0.159	0.874
Boundary			2.042	0.153
Clear	38	22		
Unclear	8	10		
Tumor shape			0.021	0.884
Regular	28	20		
Irregular	18	12		
Echoic level			0.249	0.883
Hyper-	22	16		
Iso	6	3		
Hypo-	18	13		
CDFI			0.011	0.918
Absent	4	3		
Present	42	29		
Enhancement intensity in AP			2.170	0.265
Hyper	43	32		
Non-hyper	3	0		
Vascular morphology in AP			4.070	0.044
Type 1	21	22		
Type 2	25	10		
Type 3	0	0		
Necrosis			0.055	0.815
Absent	29	21		
Present	17	11		
LR-M			3.748	0.062
Yes	10	2		
No	36	30		
AFP (µg/L)	587.41 ± 1606.26	287.84 ± 1190.72	0.763	0.448
ALT (U/L)	33.68 ± 33.56	46.58 ± 54.01	−1.285	0.203
ALB (g/L)	41.53 ± 2.81	40.43 ± 5.89	1.090	0.279
PLT (× 10^9^)	162.17 ± 49.73	167.70 ± 82.54	−0.910	0.366

*AP* arterial phase, *PLT* platelets, *ALT* alanine aminotransferase, *ALB* albumin, *AFP* alpha-fetoprotein, *LR-M* Liver Imaging Reporting and Data System M

### H-CEUS features between the recurrence group and no recurrence group

There were three features, including vascular morphology, Ki-67 expression and MVI status, that were different between the recurrence group and no recurrence group (*p* < 0.05) (Table 5). Compared to no recurrence group, the rate of vascular morphology type 2 was higher (62.5%). The rate of high expression of Ki-67 (81.25%) and MVI-positive cases (50%) were also higher in the recurrence group than no recurrence group.

**Table 5:** Image features and clinical data between recurrence and no recurrence groups

Variables	No recurrence (*n* = 62)	Recurrence (*n* = 16)	χ^2^/t	*p*-value
Age (years)	59.21 ± 9.66	61.56 ± 13.27	0.801	0.426
Gender			0.552	0.458
Male	54	15		
Female	8	1		
Size (cm)	3.82 ± 2.77	3.74 ± 1.55	−0.105	0.917
Boundary			1.269	0.260
Clear	46	14		
Unclear	16	2		
Morphology			0.008	0.929
Regular	38	10		
Irregular	24	6		
Echoic level			4.346	0.114
Hyper-	27	11		
Iso	9	0		
Hypo-	26	5		
CDFI			0.306	0.580
Absent	5	2		
Present	57	14		
Enhancement intensity in AP			0.805	0.370
Hyper	59	16		
Non-hyper	3	0		
Vascular morphology in AP			4.640	0.031
Type 1	38	5		
Type 2	24	11		
Type 3	0	0		
Enhancement intensity in PP			0.268	0.875
Hyper-	1	0		
Iso-	26	7		
Hypo-	35	9		
Enhancement intensity in LP			0.417	0.812
Hyper-	1	0		
Iso-	9	3		
Hypo-	52	13		
Necrosis			1.740	0.187
Absent	42	8		
Present	20	8		
LR-M			1.430	0.232
Yes	8	4		
No	54	12		
AFP (µg/L)	380.09 ± 1262.02	803.20 ± 2043.13	1.034	0.117
ALT (U/L)	39.22 ± 43.90	37.11 ± 40.42	−0.225	0.502
ALB (g/L)	41.16 ± 4.26	40.89 ± 4.54	−0.174	0.908
PLT (× 10^9^)	169.93 ± 67.52	154.69 ± 50.70	−0.675	0.641
Histological differentiation			1.124	0.570
Well	19	3		
Moderate	46	9		
Poor	13	4		
Ki-67 expression			4.128	0.043
Low level (≤ 10%)	29	3		
High level (> 10%)	33	13		
MVI			4.074	0.044
Absent	47	8		
Present	15	8		

*PLT* platelets, *ALT* alanine aminotransferase, *ALB* albumin, *AFP* alpha-fetoprotein, *AP* arterial phase, *PP* portal phase, *LP* late phase, *MVI* microvascular invasion

## Discussion

MVI is an important factor affecting the prognosis of HCC and is associated with early recurrence and low survival. CEUS has been used to predict MVI status in HCC before surgery. However, some studies indicated that arterial enhancement features are not related to MVI status. Interestingly, we first found that vascular morphology of HCC in AP on H-CEUS was one of the independent risk factors related to MVI status, which was not consistent with previous studies [15, 17, 30]. In addition, our study indicated that vascular morphology was one of the risk factors related to Ki-67 expression level and tumor recurrence. H-CEUS could find more details in AP by increasing the frame rate.

Vascular morphology of HCC is a clinically important feature; it was reported that different FLL have different vascular morphology [31]. Our previous study proved that H-CEUS has an advantage in detecting and displaying vascular morphology features in AP compared to CEUS [20]. In our study, there were only two vascular morphologies: type 1 and 2. Vascular morphology type 1 was a fine irregular vessel with dense and homogeneously distributed within HCC, indicating a low risk of MVI in our study. At present, the exact mechanism of MVI is complex and multifactorial, and tumor hypoxia plays an important role in the formation of MVI. The characteristics of vascular morphology type 1 allow the tumor to be well-vascularized and the intratumoral blood flow is homogeneous, the tumor is probably free from hypoxia, therefore the risk of MVI is low. However, vascular morphology type 2 is characterized by large vessels in the periphery of the tumor that are either absent or sparsely distributed within HCC and thus more prone to hypo-perfusion, resulting in hypoxia, and the risk of MVI is high.

CEUS Li-RADS has important clinical value in the diagnosis of HCC risk, and the study of the clinical application of Li-RADS is also the focus of CEUS. LR-M indicates that the tumor is malignant and not specifically HCC. However, a certain proportion of HCC showed atypical features on CEUS and were classified as LR-M [32]. Furthermore, the hypo-enhancement in the portal venous phase on CEUS suggested that the contrast agent washed out early and significantly within the tumor, which may indicate poor differentiation and high malignancy [19]. In this study, 15.4% cases of HCC were diagnosis of LR-M by H-CEUS. The proportion of cases with LR-M was higher in the MVI-positive group than in the MVI-negative group, which indicated that LR-M represents HCC has high risk of MVI. Our results are consistent with a previous study [33]. The mechanism between CEUS LR-M and MVI in HCC is not well understood, but we speculate that there may be the following reasons. Early rapid wash-out is one of the important diagnostic features of LR-M, which is associated with changes in the draining vessels of HCC. With the development of HCC, the draining vessels of the tumor also changed. The main drainage vessel changed from hepatic vein to portal vein [34], which results in the continuous enhancement of contrast agent perfusion of the liver in the portal venous phase, but no contrast agent perfusion of the tumor, and portal venous drainage vessels drain blood flow from the tumor to the surrounding liver, as a result, the tumor appears to rapidly wash out in the early portal venous phase, during which the intratumoral emboli migrate from the tumor along the draining portal vein to form MVI outside the tumor.

The irregular shape of the tumor may indicate the invasion of surrounding liver tissue or tumor envelope. These characteristics probably indicate that HCC has a risk of MVI [35]. Necrosis on H-CEUS usually suggests a high possibility of hypoxia, which is an important risk factor for MVI formation. Although the imaging features of irregular tumor morphology and necrosis were different between the MVI-positive group and the MVI-negative group, multivariate analysis indicated that they were not independent risk factors for MVI in this study. Tumor marker is also valuable in determining MVI risk. AFP can be used for the diagnosis of HCC in clinic, and the relationship between AFP and MVI has also been studied [36]. Although a previous study reported that higher AFP level is also helpful for indicating the probability of diagnosing MVI [37], our research reported that AFP was not an independent risk factor for MVI. Some studies also suggested that there was no difference in APF level between the MVI-positive group and the MVI-negative group [17, 30].

In this study, H-CEUS was also first used to evaluate the relationship between vascular morphology of HCC and Ki-67 expression. Vascular morphology type 2 indicated high Ki-67 expression, and vascular morphology type 1 indicated low Ki-67 expression. It is well known that high Ki-67 expression represents an active state of cell proliferation, which promotes new blood vessels to feed tumor growth. Therefore, different Ki-67 expression levels could promote different vascular morphology of HCC.

How to evaluate the prognosis of patients with HCC by preoperative imaging is one of the important clinical focuses. Our study proved that three features, including vascular morphology of HCC, MVI status and Ki-67 expression, were different between the recurrence group and no recurrence group. We first found that different vascular morphology of HCC revealed the different prognosis of patients with HCC. During the HCC progression, the tumor microvessels, including arterial vessel, portal vessel and drainage vessels, also changed. The morphology and distribution of vessels will change with the growth of HCC. CEUS is a real-time imaging technique, especially in arterial phase, which can show the tumor perfusion process. In this study, H-CEUS was used to increase the frame rate of images and display more details of microcirculation perfusion in arterial phase, which is helpful in observing different types of vascular morphology in HCC with different prognoses. In addition, it was well known that MVI and Ki-67 were high-risk factors related to recurrence; our study results were coherent with previous studies [11, 12, 24, 25].

This study also has some limitations. (1) Our study was retrospective and reliable data from prospective multicenter studies are still needed for the value of vascular morphology in predicting MVI for HCC. (2) The relationship between vascular morphology of HCC in AP on H-CEUS and vascular distribution on pathology is unclear and is worth investigating. (3) The relationship between vascular morphology of HCC and Ki-67 expression could be only one of the risk factors of recurrence. It could be part of a wider context (AFP level, prior treatment) and not considered as the only relevant factor [38, 39].

Generally, H-CEUS can clearly show different vascular morphology of HCC in AP. The different vascular morphology of HCC can indicate the different risk of MVI, Ki-67 expression and tumor recurrence, which provides a feasible imaging technique for clinician to judge the risk of MVI before operation, and it is helpful to adopt appropriate treatment.

## Data Availability

The data and material can be obtained from the first author or corresponding author.

## Abbreviations

AFP: Alpha-fetoprotein
ALB: Albumin
ALT: Alanine aminotransferase
AP: Arterial phase
CDFI: Color doppler flow imaging
CEUS: Contrast-enhanced ultrasound
CI: Confidence interval
FLL: Focal liver lesion
H-CEUS: High frame rate contrast-enhanced ultrasound
HCC: Hepatocellular carcinoma
Li-RADS: Liver imaging reporting and data system
MVI: Microvascular invasion
OR: Odds ratio
PLT: Platelets
